# The perils of plurality rule in democratic presidential systems: A replication and extension

**DOI:** 10.1371/journal.pone.0262026

**Published:** 2022-01-20

**Authors:** Joshua Holzer

**Affiliations:** Westminster College, Fulton, MO, United States of America; University of California San Francisco, UNITED STATES

## Abstract

Recent research suggests that country-years where presidents won their previous election with an absolute majority are more likely to be associated with high government respect for human rights, in comparison to country-years where presidents won their previous election by a mere plurality. With this follow-up article, I replicate these findings with a greatly expanded dataset, and I explore whether country-years where presidents have been elected using a majoritarian system are more likely to be associated with high government respect for human rights, in comparison to country-years where presidents have been elected using a non-majoritarian system. Ultimately, I find that not only are presidents elected with a plurality associated with comparatively lower levels of human rights respect, but so are presidents elected via a non-majoritarian system. These findings suggest that policymakers seeking to improve human rights practices may want to consider directing their efforts towards promoting electoral reform with an emphasis on mandating a minimum of a majority in order to win an election.

## Introduction

Why are some democratic systems associated with more human rights violations than others? Some of this variation can be explained by comparing parliamentary versus presidential systems. Richards and Gelleny ([1]: 517), for instance, find presidential systems “to be associated with lesser levels of respect.” However, not all presidential systems are the same, and some of the differences between them could influence government respect for human rights in different ways. For instance, in a recent paper [2], I disaggregated all presidents as being elected with either with a majority or a plurality, and then I compared how well these two categories protected human rights. Ultimately, I found “that in the years after a presidential election won by an absolute majority, states are more likely to experience an *increase* in government respect for human rights, in comparison to the years after a presidential election won by a mere plurality” ([2]: 1–2).

The purpose of this follow-up article is twofold. First, I seek to re-estimate my previous model with current data. Second, I seek to explore a proposal I made at the end of my previous paper, which is that “it may be prudent for non-majoritarian systems to consider adopting a mandatory majority rule” ([2]: 7). In the end, I find that my previous results are robust to a greatly expanded dataset. Furthermore, I also find country-years where presidents have been elected using a majoritarian system to be more likely to be associated with high government respect for human rights, in comparison to country-years where presidents have been elected using a non-majoritarian system. These findings suggest that policymakers seeking to improve human rights practices may want to consider directing their efforts towards promoting electoral reform with an emphasis on mandating a minimum of a majority in order to win an election.

## Theoretical argument

The primary goal of all presidential hopefuls is to get elected. According to Bueno de Mesquita *et al*. [3], two factors determine how presidents are chosen: the ‘selectorate’ and the ‘winning coalition’. The ‘selectorate’ refers to the proportion of a state’s population that has a formal role in selecting the president. The ‘winning coalition’ refers to the proportion of selectorate support that is needed for a president to obtain power. In systems where presidents are elected directly, the selectorate is all voting-eligible citizens. The minimum size of the winning coalition, however, is dependent upon whether the state uses a majoritarian or non-majoritarian electoral system. In the former, the minimum winning coalition is at least a majority of the selectorate that participates in the election. In the latter, the minimum winning coalition is simply more selectorate support than any other candidate.

Bueno de Mesquita *et al*. ([3]: 8) argue that “[i]n political systems characterized by small winning coalitions and large selectorates—as is common in many rigged-election autocracies—supporters of the leader are particularly loyal because the risk and cost of exclusion if the challenger comes to power are high.” In such systems, it is more efficient for an autocrat to depend upon the use of private goods to earn (and maintain) the support and loyalty of their winning coalition. However, when the minimum winning coalition is large, it becomes prohibitively expensive to provide private goods to each individual member of the leader’s alliance. In such cases, public goods are used to try and win over (and later satisfy) a winning coalition. Incidentally, public goods benefit the public at large, including those not even in the leader’s alliance.

Recently, human rights scholars have begun arguing that government respect for human rights could (and should) be viewed as a public good [4, 5]. Returning to Bueno de Mesquita *et al*. ([3]: 351), they find that “[r]espect for human rights is common in systems with large winning coalitions,” while “oppression of political opponents is common in systems with small winning coalitions,” which lends support to the suggestion that government respect for human rights could/should be viewed as a public good. Noting that “inclusive, large-coalition polities tend to produce the most public benefits” ([3]: 485), one would presume that presidents able to build large winning coalitions would be more apt to provide public goods, and by extension, protect human rights. This leads me to my first hypothesis, which is actually a restatement of that which was used in my previous paper [2]:

1. I argue that country-years where presidents won their previous election with an absolute majority are more likely to be associated with high government respect for human rights, in comparison to country-years where presidents won their previous election by a mere plurality.

The study of political coalitions in parliamentary systems has long been a focus of scholarly interest [6]. Recently, however, political coalitions in presidential systems have become an active research area [7–20]. Freudenreich ([21]: 82), for instance, links “governability in presidential systems…with the ability of presidents to form and manage cabinet coalitions.” One of the major differences between majoritarian and non-majoritarian elections can “be found in the different incentives they provide for coalition-making among parties” ([22]: 116); the former incentivizes candidates to broaden their coalition in order to obtain majority support, while the latter only encourages candidates to ensure that their coalition is larger than their competitors (which may not necessarily represent a majority of the voting selectorate). I argue that this difference has human rights repercussions, as research shows that when presidents build broad, inclusive coalitions, their administrations are less likely (and less able) to trample on human right [23], which is consistent with the conclusion of Bueno de Mesquita *et al*. ([3]: 461) that “social welfare is enhanced when leaders depend on a large coalition.” Given that majoritarian electoral systems mandate large coalitions, one would except such systems to be better at protecting human rights versus those systems that allow for much smaller minimum winning coalitions. This leads me to my second hypothesis:

2. I argue that country-years where presidents were elected using a majoritarian system are more likely to be associated with high government respect for human rights, in comparison to country-years where presidents were not elected using a majoritarian system.

## Methods

### Sample

Recall that the purpose of this article is the replicate and extend upon the findings of my previous paper [2]. As such, my sample for this follow-up article is modeled on that which was used in my previous paper—i.e. presidential democracies with ‘democracy’ defined using the Democracy versus Dictatorship (DD) dataset [24]. Per the DD dataset, a regime is considered to be a ‘democracy’ when the president is elected, the legislature is elected, there is more than one party competing in elections, and an alternation under identical electoral rules has taken place ([24]: 69).

### Dependent variable

As was the case for my previous paper [2], my dependent variable for this follow-up article is the nine-point Physical Integrity Rights Index (henceforth ‘PHYSINT scores’) developed by Cingranelli and Richards [25]. PHYSINT scores are created by adding together four constituent indicators of government respect for human rights (i.e. the rights of all human beings to be protected from political imprisonment, torture, disappearance, and extrajudical killing), which in turn are derived from U.S. State Department and Amnesty International annual reports. PHYSINT scores are ordinal and range from ‘0’ (which suggests no respect for any of the four constituent indicators of government respect for human rights for that particular country-year) to ‘8’ (which suggests full respect for all four constituent indicators for that particular country-year). While my previous paper [2] utilized Version 2014.04.14 of the data [26], for this follow-up article I use Version 2021.01.21 [27], which provides substantially more coverage.

Despite widespread use, in recent years PHYSINT scores have had to contend with some amount of criticism. Clark and Sikkink ([28]: 567), for instance, argue that “[b]ecause of [the] increased quality and quantity of information,” PHYSINT scores “may skew toward worse scores in later years.” Fariss ([29]: 300) suggests that this is because over time “the U.S. State Department and Amnesty International look harder for abuse, look in more places for abuse, and classify more acts as abuse,” which he calls “a *changing standard of accountability*” (297). In an attempt to address this, Fariss created latent scores for human rights which he claims are “unbiased estimates of repression” ([29]: 297). Since this point, some scholars have followed Fariss in abandoning the use PHYSINT scores.

More recently, Haschke and Gibney ([30]: 89–90) point out that while Clark and Sikkink [28] and Fariss [29] “repeatedly assert that the [U.S. State Department and Amnesty International] annual reports are now longer and more detailed—and presumably more accurate—than they had been in the past,” their “evidence…is selective and does not constitute compelling proof.” Indeed, Haschke and Gibney ([30]: 99) suggest that “any measurable bias is actually in the opposite direction of” what Clark and Sikkink [28] and Fariss [29] claim. In another critique, Cingranelli and Filippov ([31]: 1088) identify “serious problems” with the supposedly ‘unbiased estimates’ that Fariss has created. They demonstrate that the upward trend identified by Fariss “depend[s] almost entirely on the inclusion of the mass killing indicators” ([31]: 1086). As such, Cingranelli and Filippov ([31]: 1088) caution that “[t]hose who use Fariss’s scores should be aware that there is a strong built-in correlation between mass killings and those scores,” and therefore, “evaluators should remember that the trends in Fariss’s scores for capable and democratic countries are affected by frequencies of mass killing events in failed and authoritarian states.” Cingranelli and Filippov ([31]: 1089) bluntly argue that Fariss’s “latent scores should not be used as dependent variables in conventional regression analysis.” They go so far as to suggest that in creating his latent measure, Fariss’s efforts “comes close to data manufacture” ([32]: 274). With their latest co-authored piece, Cingranelli and Filippov sow further doubts in the validity of Fariss’s scores [33].

Given the robust criticism, which I have reviewed elsewhere [34], it would be improper for me to use Fariss’s latent scores as my sample is limited to democracies, and as such, I do not want my results to be influenced by the high rate of human rights violations seen in nondemocratic authoritarian contexts. To conclude, I would like to point out that the use of PHYSINT scores has not completely fallen out of fashion; in 2021 alone, there have been several human rights-related papers published that have opted to use these scores despite the creation of Fariss’s latent measure [23, 35–37].

### Independent variables

In order to test my two hypotheses, I have constructed the following two primary independent variables: *president elected with a majority* and *president elected using a majoritarian system*. While the latter is entirely new to this follow-up article, the former is a recreation of that which was used in my previous paper [2]. For that previous paper, I utilized Version 2.0 of the Democratic Electoral Systems Around the World dataset [38]; for this follow-up article, I utilized Version 3.0 [39]. For each country-year, I looked at the most recent democratic election which brought the current president to power. If the president had been elected with more than 50% of the vote, my *president elected with a majority* variable was coded as ‘1’. If the president had been elected with less than 50% of the vote, my variable was coded as ‘0’. For my new *president elected using a majoritarian system* variable, I also looked at the most recent democratic election which brought the current president to power. In this case, if the president had been elected using an electoral system that required more than 50% of the vote, my *president elected using a majoritarian system* variable was coded as ‘1’. Otherwise, if the president had been elected using an electoral system that did not required more than 50% of the vote, my variable was coded as ‘0’.

As was the case for my previous paper [2], all country-years where the president was not directly elected were omitted from my analysis. Note that for country-years that were election years, I coded that year based on which president (i.e. either the outgoing or incoming) presided for the majority of that year (for my *president elected with a majority* variable) and which electoral system had been in effect for the majority of that year (in the rare circumstances that a country switched between majoritarian and non-majoritarian systems). An overview of all the countries in my sample, as well as which had at least one president elected with a majority, which had at least one president elected without a majority, which had at least one presidential election that used a majoritarian system, and which had at least one presidential election that did not use not a majoritarian system can be seen in Table 1.

**Table 1:** Democratic presidential systems with directly-elected presidents, 1990-2017.

Argentina^⊙^^⊗^^⊠^	Armenia^⊙^^⊡^	Austria^⊙^^⊡^	Benin^⊙^^⊡^	Bolivia^⊙^^⊗^^⊡^^⊠^
Brazil^⊙^^⊡^	Bulgaria^⊙^^⊡^	Chile^⊙^^⊡^	Colombia^⊙^^⊡^	Costa Rica^⊙^^⊗^^⊠^
Croatia^⊙^^⊡^	Cyprus^⊙^^⊡^	Dominican Republic^⊙^^⊗^^⊡^^⊠^	Ecuador^⊙^^⊡^^⊠^	El Salvador^⊙^^⊡^
Finland^⊙^^⊡^	France^⊙^^⊡^	Georgia^⊙^^⊡^	Ghana^⊙^^⊡^	Guatemala^⊙^^⊡^
Guinea-Bissau^⊙^^⊡^	Honduras^⊙^^⊗^^⊠^	Indonesia^⊙^^⊡^	Ireland^⊙^^⊡^	Kenya^⊙^^⊗^^⊡^^⊠^
Republic of Korea^⊙^^⊗^^⊠^	Kyrgyz Republic^⊙^^⊡^	Liberia^⊙^^⊡^	Lithuania^⊙^^⊡^	Madagascar^⊙^^⊡^
Malawi^⊙^^⊗^	Mali^⊙^^⊡^^⊠^	Mexico^⊗^^⊠^	Mongolia^⊙^^⊡^	Nicaragua^⊙^^⊗^^⊠^
Niger^⊙^^⊡^	Nigeria^⊙^^⊡^	North Macedonia^⊙^^⊡^	Panama^⊙^^⊗^^⊠^	Paraguay^⊗^^⊠^
Peru^⊙^^⊡^	Philippines^⊗^^⊠^	Poland^⊙^^⊡^	Portugal^⊙^^⊡^	Romania^⊙^^⊡^
Senegal^⊙^^⊡^	Serbia^⊙^^⊡^	Sierra Leone^⊙^^⊡^	Slovak Republic^⊙^^⊡^	Slovenia^⊙^^⊡^
Sri Lanka^⊙^^⊠^	Timor-Leste^⊙^^⊡^	Ukraine^⊙^^⊡^	Uruguay^⊙^^⊗^^⊡^^⊠^	Venezuela^⊙^^⊗^^⊠^

^⊙^At least one president was elected with a majority

^⊗^At least one president was elected without a majority

^⊡^At least one presidential election used a majoritarian system

^⊠^At least one presidential election did not use not a majoritarian system

Beyond my two primary independent variables, I include the same control variables I used in my previous paper [2], albeit more recent versions where applicable. For instance, I use an *executive constraint* variable, which takes into account “the extent of institutionalized constraints on the decision-making powers of chief executives” ([40]: 24). In my previous paper, I used the PolityIV [41] version of this variable, whereas in this follow-up article, I use the POLITY5 version. Next, (logged) measures of gross domestic product (GDP) per capita and population size, both of which are from the World Bank [42]. While my previous paper included a measure for *domestic conflict* drawn from Version 1-2005 of the the UCDP/PRIO Armed Conflict Dataset [43], I now use Version 20.1 [44]. This variable is coded as ‘0’ for each country-year that experienced fewer than than 25 battle-related fatalities, ‘1’ for each country-year that experienced at least 25 but less than 1,000 battle-related fatalities, and ‘2’ for each country-year that experienced at least 1,000 battle-related fatalities. Finally, following my previous paper (and others [23, 34, 45–47]), I include a dummy variable for each lagged level of the dependent variable (excluding the most repressive category) in order to account for “[p]atterns of abuse [that] tend to persist over time” ([3]: 352). Summary statistics for my dependent variable and all control variables are reported in Table 2. These statistics have been reported separately for each category of my two primary independent variables (i.e. *president elected with a majority* and *president elected using a majoritarian system*) and well as for all country-years.

**Table 2:** Summary statistics.

Country-years where the president was elected with a majority							
	Obs	Countries	Min	Mean	Mode (Freq)	Max	Std Dev
PHYSINT score^1^	979	52	0	5.385	7 (232)	8	1.899
Executive constraint^2^	979	52	3	6.147	7 (475)	7	0.972
(Logged) GDP per capita	979	52	5.771	8.426	—	11.181	1.325
(Logged) population size	979	52	13.550	16.139	—	19.394	1.217
Domestic conflict^3^	979	52	0	0.105	0 (900)	2	0.379
Country-years where the president was not elected with a majority							
	Obs	Countries	Min	Mean	Mode (Freq)	Max	Std Dev
PHYSINT score^1^	203	15	0	4.635	7 (41)	8	1.981
Executive constraint^2^	203	15	4	6.182	6 (112)	7	0.690
(Logged) GDP per capita	203	15	5.761	8.297	—	10.222	1.045
(Logged) population size	203	15	14.823	16.607	—	18.642	1.245
Domestic conflict^3^	203	15	0	0.118	0 (180)	2	0.339
Country-years where the president was elected using a majoritarian system							
	Obs	Countries	Min	Mean	Mode (Freq)	Max	Std Dev
PHYSINT score^1^	836	43	0	5.580	7 (223)	8	1.847
Executive constraint^2^	836	43	3	6.261	7 (443)	7	0.903
(Logged) GDP per capita	836	43	5.771	8.498	—	11.181	1.353
(Logged) population size	836	43	13.550	16.125	—	19.394	1.272
Domestic conflict^3^	836	43	0	0.093	0 (771)	2	0.340
Country-years where the president was not elected using a majoritarian system							
	Obs	Countries	Min	Mean	Mode (Freq)	Max	Std Dev
PHYSINT score^1^	346	17	0	4.474	5 (76)	8	1.916
Executive constraint^2^	346	17	3	5.893	6 (148)	7	0.943
(Logged) GDP per capita	346	17	5.761	8.176	—	10.222	1.057
(Logged) population size	346	17	14.720	16.447	—	18.642	1.105
Domestic conflict^3^	346	17	0	0.142	0 (309)	2	0.438
All country-years							
	Obs	Countries	Min	Mean	Mode (Freq)	Max	Std Dev
PHYSINT score^1^	1,182	55	0	5.256	7 (273)	8	1.933
Executive constraint^2^	1,182	55	3	6.153	7 (541)	7	0.930
(Logged) GDP per capita	1,182	55	5.761	8.404	—	11.181	1.282
(Logged) population size	1,182	55	13.550	16.219	—	19.394	1.234
Domestic conflict^3^	1,182	55	0	0.107	0 (1080)	2	0.372

^1^Higher values indicate greater government respect for human rights.

^2^Higher values indicate greater constraints on presidential power.

^3^0 indicates fewer than than 25 battle-related fatalities, 1 indicates at least 25 but less than 1,000 battle-related fatalities, and 2 indicates at least 1,000 battle-related fatalities.

### Model specification

Given that PHYSINT scores are *ordinal*, and given that they are being using as my dependent variable, the use of linear regression would be inappropriate, as it would lead to biased inferences, which I have discussed elsewhere [48]. Long and Freese ([49]: 309) explain that although it might be “tempting to analyze ordinal outcomes with the linear regression model (LRM)…an ordinal dependent variable violates the assumptions of LRM, which can lead to incorrect conclusions.” In place of using a linear regression model, Long and Freese point to McKelvey and Zavoina [50] who pioneered the use of ordered probit models. In light of Long and Freese’s advice, both of my models below utilize ordered probit regression. Of note, my previous paper [2]—which this follow-up article is intended to build upon—also estimated ordered PHYSINT scores by using ordered probit regression, which is consistent with the broader human rights literature [1, 23, 34, 51–54].

### Results

In Table 3, you can see two ordered probit models that estimate PHYSINT scores in presidential democracies. To address the pooled nature of the data, as well as heteroscedasticity, both of these models have robust standard errors clustered by country, which is in line with the human rights literature [2, 23, 34, 53, 55]. Before looking to my primary independent variables, I want point out that across both models all control variables are statistically significant and have signs pointing in the expected direction. For instance, *executive constraint* and *(logged) GDP per capita* are both found to be positively associated with high PHYSINT scores, while *(logged) population size*, *domestic conflict*, and the dummy variables for the lagged levels of the dependent variable are all found to be negatively associated with high PHYSINT scores. Each of these variables are found to statically significant at least at the 95% level for both models.

**Table 3:** Ordered probit estimates of government respect for human rights in presidential democracies, 1990-2017.

	Model 1	Model 2
President elected with a majority	0.207*	
	(0.092)	
President elected using a majoritarian system		0.257**
		(0.093)
Executive constraint	0.162**	0.142**
	(0.053)	(0.052)
(Logged) GDP per capita	0.126*	0.131*
	(0.053)	(0.051)
(Logged) Population size	-0.209**	-0.220**
	(0.051)	(0.054)
Domestic conflict	-0.801**	-0.803**
	(0.095)	(0.094)
PHYSINT score at 0 in the previous year	-4.865**	-4.830**
	(0.529)	(0.543)
PHYSINT score at 1 in the previous year	-4.394**	-4.357**
	(0.376)	(0.382)
PHYSINT score at 2 in the previous year	-3.741**	-3.686**
	(0.330)	(0.323)
PHYSINT score at 3 in the previous year	-3.369**	-3.303**
	(0.304)	(0.302)
PHYSINT score at 4 in the previous year	-2.631**	-2.600**
	(0.264)	(0.269)
PHYSINT score at 5 in the previous year	-2.001**	-1.968**
	(0.196)	(0.193)
PHYSINT score at 6 in the previous year	-1.462**	-1.443**
	(0.193)	(0.195)
PHYSINT score at 7 in the previous year	-0.823**	-0.814**
	(0.155)	(0.157)
Number of observations	1,182	1,182
Number of countries	55	55

* p < 0.05,

** p < 0.01. Numbers in parentheses are robust standard errors clustered by country.

Looking specifically to Model 1, recall that my first hypothesis is that country-years where presidents won their previous election with an absolute majority are more likely to be associated with high government respect for human rights, in comparison to country-years where presidents won their previous election by a mere plurality. Again, note that this hypothesis is actually a restatement of that which was used in my previous paper [2]. The purpose of Model 1 is to re-estimate my previous model so as to ascertain whether my previous findings are robust to the new data that has become available in the last few years. My original model [2] included 241 observations from 35 countries over an 11-year period. Model 1 in Table 3 includes 1,182 observations from 55 countries over an 28-year period. As you can see, my *president elected with a majority* variable is found to be positively associated with high PHYSINT scores, and this relationship is statistically significant at least at the 95% level. In other words, my previous findings are found to be robust to a greatly expanded dataset.

Moving now to Model 2, recall that my second hypothesis is that country-years where presidents were elected using a majoritarian system are more likely to be associated with high government respect for human rights, in comparison to country-years where presidents were not elected using a majoritarian system. Again, the purpose of this model is to explore whether “majoritarian elections [do] indeed promote greater government respect for human rights” ([2]: 7), which I put forward at the end of my previous paper. As you can see, my *president elected using a majoritarian system* variable is found to be positively associated with high PHYSINT scores, and this relationship is statistically significant at least at the 99% level.

Although the results reported in Table 3 are encouraging, the effects of both models can more meaningfully be explained by analyzing predicted probabilities, a technique I have used elsewhere [56, 57]. In Table 4, I report predicted probabilities (and 95% confidence intervals) for PHYSINT scores when presidents are elected with a majority or using a majoritarian system. These probabilities were estimated using a Stata module designed by Tomz, Wittenberg, and King [58]. All probabilities have been derived from the means and modes of my control variables (depending upon whether the variables were continuous in the case of the former or categorical in the case of the latter); these values can be seen in the bottom fifth of Table 2 (i.e. under ‘All country-years’). Note that for space considerations, the probabilities of PHYSINT scores below 4 were not reported, as they were all near-zero, which makes sense as democratic states typically do not engage in such high levels of repression.

**Table 4:** Predicted probabilities of government respect for human rights when presidents are elected with a majority or using a majoritarian system.

	PHYSINT score				
	4	5	6	7	8
Not elected with a majority	0.025	0.145	0.296	0.436	0.095
	[0.013, 0.043]	[0.100, 0.198]	[0.254, 0.338]	[0.359, 0.512]	[0.054, 0.149]
Elected with a majority	0.016	0.108	0.263	0.477	0.135
	[0.007, 0.030]	[0.070, 0.155]	[0.216, 0.310]	[0.396, 0.555]	[0.080, 0.204]
Difference	-0.009	-0.037	-0.033	0.041	0.040
	[-0.019, -0.001]	[-0.071, -0.005]	[-0.066, -0.004]	[0.005, 0.081]	[0.005, 0.080]
Percentage change	-37.1%	-25.3%	-11.3%	9.4%	41.6%
	PHYSINT score				
	4	5	6	7	8
Not a majoritarian system	0.028	0.154	0.304	0.426	0.086
	[0.014, 0.047]	[0.104, 0.211]	[0.259, 0.348]	[0.344, 0.508]	[0.049, 0.136]
Majoritarian system	0.015	0.107	0.264	0.479	0.134
	[0.007, 0.029]	[0.070, 0.152]	[0.217, 0.310]	[0.400, 0.555]	[0.090, 0.202]
Difference	-0.012	-0.046	-0.040	0.053	0.047
	[-0.024, -0.003]	[-0.084, -0.013]	[-0.072, -0.011]	[0.014, 0.096]	[0.013, 0.089]
Percentage change	-43.9%	-30.2%	-13.2%	12.4%	54.9%

95% confidence intervals are in brackets.

Starting with the top-half of Table 4, you can see that the probability of a PHYSINT score of ‘8’ (which suggests full respect for government respect for human rights) is 0.095 when the president is not elected with a majority. In comparison, note that the probability of a PHYSINT score of ‘8’ is 0.135 when the president is elected with a majority. The difference in the probability going from 0.095 to 0.135 is 0.040, and this difference is statistically significant at least at the the 95% level, given that the corresponding 95% confidence interval (which is in brackets) does not overlap with zero. While 0.040 may not seem like much, note that increasing a 0.095 probability by 0.040 is roughly a 42% increase. Put another way, this means that for a given year, the ‘average’ state in my dataset (i.e. a state whose parameters match the mean/mode of my control variables for all country-years, which can be seen in the bottom fifth of Table 2) is roughly 42% *more* likely to be at the highest (PHYSINT) level of government respect for human rights when the president has been elected with a majority versus without a majority.

Moving along, you can see that a PHYSINT score of 7 corresponds with a positive percent change (i.e. roughly 9%), while lower PHYSINT scores (such as ‘4’ through ‘6’) all correspond with *negative* percent changes. Jointly, this suggests that a state is *more* likely to be at *higher* levels of government respect for human rights and *less* likely to be at *lower* levels of government respect for human rights when the president has been elected with a majority versus without a majority. In other words, consistent with my first hypothesis (as well as my previous paper [2]), presidents elected with a majority appear to be better at protecting human rights than presidents elected without a majority. Looking at the bottom half of Table 4, this trend carries over to presidents elected via a majoritarian system which is consistent with my second hypothesis, as states are *more* likely to be at *higher* levels of government respect for human rights and *less* likely to be at *lower* levels of government respect for human rights when presidents have been elected using a majoritarian system versus a non-majoritarian system.

## Conclusion

According to Macedo ([59]: 1038), majoritarian systems are “flawed,” as “[m]ajority rule says that the loss for the few is justified by the fact that the winners are greater in number.” Writing along similar lines more than 150 years ago, Mill ([60]: 76) warns of “the tyranny of the majority.” Despite this iconic phrase being closely associated with Mill, he did not coin it, but rather borrowed it from Tocqueville ([61]: 306), who also feared the “tyranny of the majority” ([62]: 195). Even Tocqueville, though, likely did not conceive of this concept, as some of his writings bear a strikingly resemblance to Aristotle [63], who cautioned against the “principle that the multitude ought to be supreme rather than the few” ([64]: 121). However, not all contemporary scholars agree with these ideas. McGann ([65]73-74), for instance, “argue[s] that…majority rule provides more protection for the worst-off minority than any other decision rule.” McGuire and Olson ([66]: 95) note that the “majority, even when it thinks only of itself and has no concern for the losses of the minority…treats the minority as well as it treats itself.” Finally Bueno de Mesquita *et al*. ([3]: 351) find that “[s]ystems with large winning coalitions engage in substantially less oppression than those with small winning coalitions.”

Building upon this literature, in a recent paper, I found that country-years where presidents won their previous election with an absolute majority are more likely to be associated with high government respect for human rights, in comparison to country-years where presidents won their previous election by a mere plurality. In the end, I conclude that “one of presidentialism’s greatest perils is the plurality election” ([2]: 7). With this follow-up article I have replicated my previous findings and have found them to be robust to a greatly expanded dataset. I have also found country-years where presidents have been elected using a majoritarian system to be more likely to be associated with high government respect for human rights, in comparison to country-years where presidents have been elected using a non-majoritarian system. Put differently, not only are presidents elected with a plurality associated with comparatively lower levels of human rights respect, but so are presidents elected via a non-majoritarian system. While, “[n]o rule is a panacea” ([67]: 347), for policymakers seeking to improve human rights practices, promoting electoral reform with an emphasis on mandating a minimum of a majority may increase the likelihood that human rights protecting presidents will be elected.
